# Responses of Retinal Ganglion Cells to Extracellular Electrical Stimulation, from Single Cell to Population: Model-Based Analysis

**DOI:** 10.1371/journal.pone.0053357

**Published:** 2012-12-28

**Authors:** David Tsai, Spencer Chen, Dario A. Protti, John W. Morley, Gregg J. Suaning, Nigel H. Lovell

**Affiliations:** 1 Graduate School of Biomedical Engineering, University of New South Wales, Sydney, New South Wales, Australia; 2 Howard Hughes Medical Institute, Department of Biological Sciences, Columbia University, New York, New York, United States of America; 3 Bioelectronic Systems Lab, Department of Electrical Engineering, Columbia University, New York, New York, United States of America; 4 Discipline of Physiology and Bosch Institute, University of Sydney, Sydney, New South Wales, Australia; 5 School of Medicine, University of Western Sydney, Sydney, New South Wales, Australia; Dalhousie University, Canada

## Abstract

Retinal ganglion cells (RGCs), which survive in large numbers following neurodegenerative diseases, could be stimulated with extracellular electric pulses to elicit artificial percepts. How do the RGCs respond to electrical stimulation at the sub-cellular level under different stimulus configurations, and how does this influence the whole-cell response? At the population level, why have experiments yielded conflicting evidence regarding the extent of passing axon activation? We addressed these questions through simulations of morphologically and biophysically detailed computational RGC models on high performance computing clusters. We conducted the analyses on both large-field RGCs and small-field midget RGCs. The latter neurons are unique to primates. We found that at the single cell level the electric potential gradient in conjunction with neuronal element excitability, rather than the electrode center location *per se*, determined the response threshold and latency. In addition, stimulus positioning strongly influenced the location of RGC response initiation and subsequent activity propagation through the cellular structure. These findings were robust with respect to inhomogeneous tissue resistivity perpendicular to the electrode plane. At the population level, RGC cellular structures gave rise to low threshold hotspots, which limited axonal and multi-cell activation with threshold stimuli. Finally, due to variations in neuronal element excitability over space, following supra-threshold stimulation some locations favored localized activation of multiple cells, while others favored axonal activation of cells over extended space.

## Introduction

Neural prosthetic devices using electric pulses to stimulate the central nervous system have been used to restore motor function [1] and auditory percepts [2] in disabled individuals. Electrical stimulation of the retina has also become a promising strategy for restoring sight to the blind [3]–[6]. The effects of electrical stimulation on the central nervous system have been studied through both experimental [7]–[11] and theoretical approaches [12], [13]. For neurons in the brain, at the single-cell level, extracellular stimuli activate neuronal elements within a small volume around the electrode [10]. Within this volume, highly excitable elements such as the axon (in particular, the axon initial segment and the nodes of Ranvier) are preferentially recruited [9], [14]. At the population-level, such a pattern of excitation would presumably elicit sparse but spatially extended activation of neurons. This hypothesis is supported by a recent imaging study of activity in a cortical neuronal population following electrical microstimulation [7]. Following progression of neurodegenerative diseases, the retinal ganglion cells (RGCs) continue to survive in large numbers [15]. Similar to cortical neurons, these cells could be stimulated electrically to elicit responses [16]–[22]. However, the retinal neural-anatomical structure is considerably different from the brain. In particular, the axons of RGCs are unmyelinated in the eye between the soma and the optic disk, and run along a plane over the retinal inner surface. Therefore neurophysiological findings from the brain may not readily generalize to the retina.

It is not clear how the RGCs respond to extracellular electrical stimulation at the sub-cellular level and how this influences the whole-cell response. It has also proven elusive to determine the extent of axonal stimulation, and more broadly, how this might affect the number and extent of RGCs activated as a population. Clinical investigations have reported variable percepts ranging from a small spot to an oval, or sometimes a line [5], [23], [24]. These latter percepts were thought to be a consequence of spatially extensive neuronal recruitment through passing axons. In addressing this issue, electrophysiological studies have yielded conflicting results. Some studies reported focal activation of single cells (of the same type) [18], [25], while others found evidence of extensive passing axon activation [17], [26].

Taking advantage of the computational power of high performance computing clusters and recent advances in techniques for large-scale simulations of morphologically and biophysically detailed neuronal models [27]–[29], we began by examining how individual RGCs respond to electrical stimulation under a variety of stimulus conditions. Notably, we found the threshold to be strongly determined by the electric potential gradient in relation to the neuronal elements, rather than the electrode center position *per se*. Next, we extended the analyses to a population of RGCs. Here the cells formed low threshold hotspots, thereby promoting focal activation when stimulating at threshold. Following supra-threshold stimulation, some locations favored activation over extended space, while others favored localized activation. The responses predicted at the population level can explain the more complex types of percepts reported in clinical studies and suggest that the neurons’ morphology and biophysics, in addition to the electric potential of the artificial stimuli, strongly influence the responses elicited.

## Results

### The Model Reproduces Biological Observations

**Figure 1:**
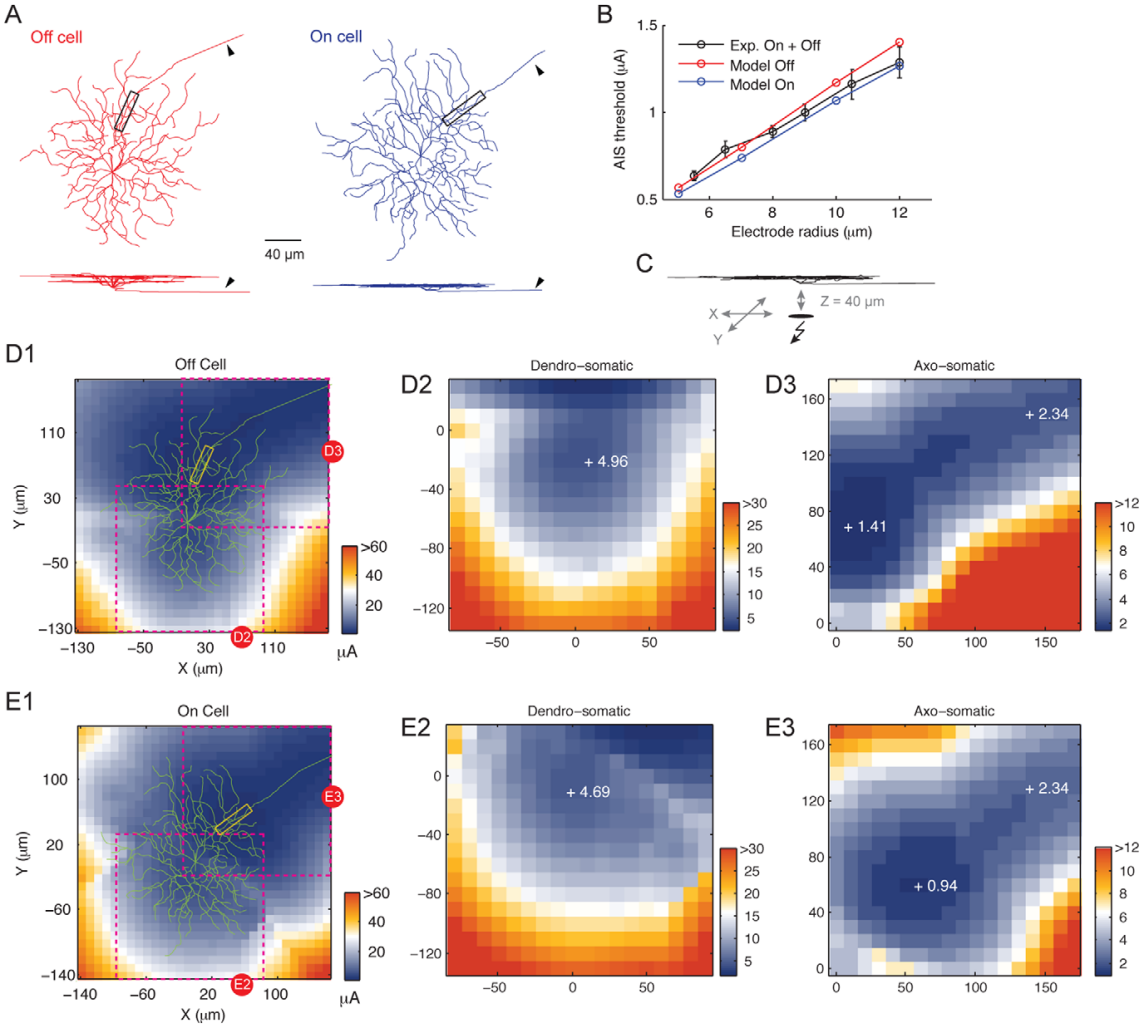
Morphology and threshold of large-field RGCs. (A) Top and side skeletal view of the On and Off large-field RGCs. Triangles indicate axons. Boxed regions indicate AIS. These cells were traced from mice retinas. (B) Comparison of the model neurons’ AIS thresholds to experimental results under equivalent conditions. The disk electrode was 40 µm axially above the cells’ somatic center. (C) Illustration of the threshold mapping procedure. A 10 µm radius disk electrode was moved through a 2D plane 40 µm axially above the somatic center. (D1) Threshold map of the Off RGC and expanded view of the dendro-somatic region (D2) and axo-somatic region (D3). (E1) Threshold map of the On RGC and expanded view of the dendro-somatic region (E2) and axo-somatic region (E3). Number next to each+symbol represents the threshold (in µA) at the marked location.

Retinal ganglion cells (RGCs) survive in large numbers following neurodegenerative diseases [15]. These cells could be stimulated by extracellular electrical pulses to produce visual percepts in the blind [4]–[6], [24]. To examine how the anatomically complex RGC neuronal elements respond to extracellular electrical stimulation, we constructed morphologically and biophysically detailed models of large-field mammalian On and Off RGCs (Figure 1A). The biophysics was based on formalizations that reproduced a wide range of experimental observations [30], [31]. To ascertain that the model neurons also adequately captured biological behaviors following extracellular electrical stimulation, we compared their stimulation threshold to experimental results. The threshold is defined as the lowest stimulus current that elicited an RGC action potential. We delivered a cathodic-first biphasic stimulus via a disk electrode from the vitreous-side 40 µm axial distance above the RGC somatic center. We determined the axon initial segment (AIS) threshold of the model RGCs by taking the mean threshold over three locations along the AIS: the proximal end, the mid-point and the distal end. The model RGCs closely reproduced the experimental observations [25] under comparable conditions over a range of electrode sizes (Figure 1B).

### Spike Elicitation Threshold along the RGC Compartmental Structure

For all electrode sizes, the modeled responses of On cells have a slightly lower threshold than those of Off cells, consistent with experimental results [18]. What might account for this difference despite otherwise identical biophysics in the model cells? First, we checked if the lower stratification depth of the On cell dendrites (Figure 1A), and thus closer proximity to the stimulating electrode, may underlie the lower threshold. We removed the dendritic tree of both RGCs, thereby eliminating dendritic variations. This had little effect on the AIS threshold for both cell types. Close examination of the morphology revealed that the On cell proximal axon (including the AIS) was approximately 2 µm closer to the stimulating electrode than the Off cell axon. Thus, while leaving the dendritic processes of both cells unperturbed, we moved the first 100 µm of the Off cell proximal axon (including the AIS) 2 µm closer to the electrode, to match the On cell. After this manipulation the thresholds of these two cell types were comparable. Together, these data suggest that the On and Off cell AIS threshold differences could be strongly influenced by the axonal location. The dendrites played little role in this, despite their total volume and extensive spatial coverage.

To examine how the stimulating electrode position affects the threshold, we mapped the RGCs’ threshold over a two-dimensional plane with a 10 µm electrode (Figure 1C). For both the Off (Figure 1D1) and On (Figure 1E1) cell the region of lowest threshold aligned with the AIS (Figure 1D3 and 1E3, c.f. boxed region in Figure 1D1 and 1E1) and the axons also formed a low threshold corridor. These results are consistent with experimental observations [32] and recent theoretical predictions [33]. Less well understood is the RGC threshold around the dendro-somatic axis. This area also encompassed a region of relatively low threshold. However, for both RGC types the minimum threshold of the dendritic region was always higher than the AIS and the axon proper (Figure 1D2 and 1E2).

### The AIS has the Lowest Threshold in Inhomogeneous Tissue

**Figure 2:**
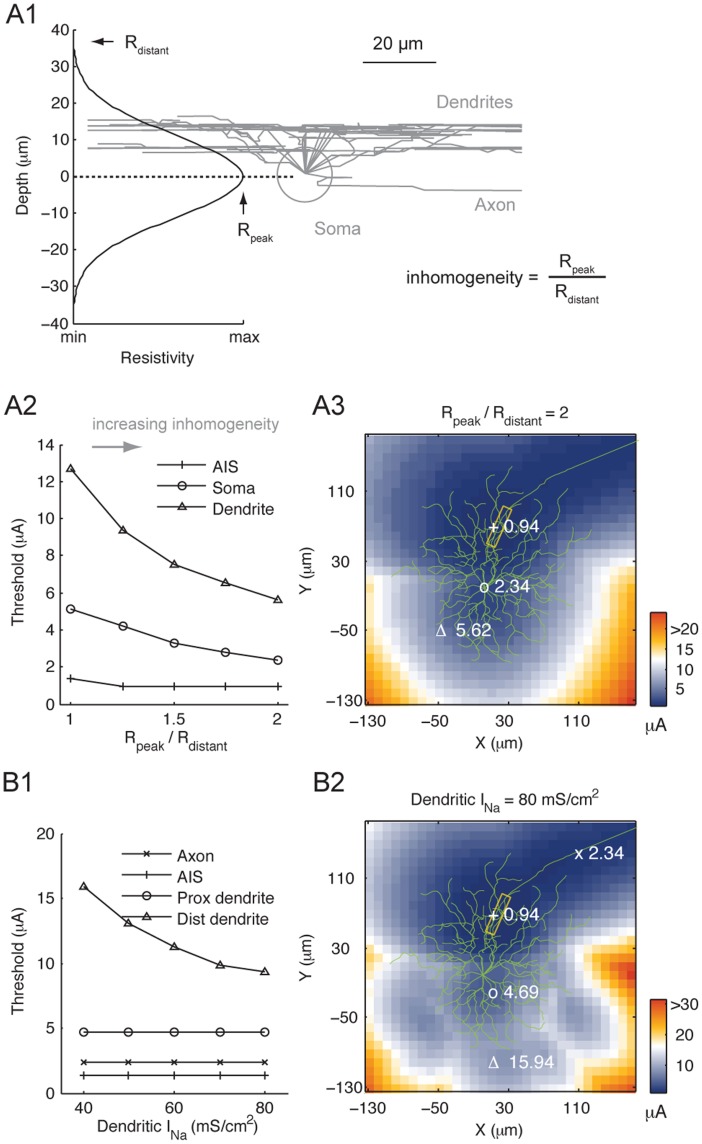
The influences of tissue resistance inhomogeneity and dendritic excitability on RGC threshold. The stimulation electrode had 10 µm radius. (A1) Variation of tissue resistivity as a function of retinal depth. Peak resistivity occurred at the center of RGC somatic layer. (A2) Threshold variation of cellular locations, marked in (A3), with increasing tissue resistance inhomogeneity. (A3) Threshold map for the Off RGC when the maximum (R_peak_) and minimum (R_distant_) resistivity differed by a factor of 2. Number next to the symbols (+, o and Δ) designates threshold (in µA) at the marked location. The shape of the threshold map is comparable to those with homogeneous tissue. In particular, the AIS (boxed region) continued to have the lowest threshold. (B1) Increasing dendritic I_Na_ reduced the threshold of distal dendrites, but had little effect on the threshold of other cellular components. The default conductance used in this study is 40 mS/cm^2^. (B2) Threshold map of the Off RGC with 80 mS/cm^2^ dendritic I_Na_ (twice the value used throughout this study).

The retinal resistivity was homogeneous in the foregoing analyses. It has been suggested that the somatic layer may have up to twice the resistivity of the dendritic and axonal layers [34], [35]. We examined how such inhomogeneity might affect RGC threshold. We varied the stimulation transfer resistivity by an axial-distance-dependent scaling factor, which peaked at the RGC somatic layer and reduced with increasing distance from this region (Figure 2A1). The extent of inhomogeneity in the direction perpendicular to the electrode is succinctly expressed as a ratio of resistivity at the somatic layer (R_peak_) over resistivity at a distant depth level (R_distant_). Increasing the inhomogeneity reduced the response threshold (Figure 2A2). The reduction was most apparent for the distal dendrites while minimal at the AIS. Nonetheless, the AIS always had the lowest threshold. The threshold map also resembled that of homogeneous tissue (Figure 1D1), even at the highest inhomogeneity tested (Figure 2A3).

The dendrites of direction selective RGCs (DSGCs) are capable of firing sodium-dependent spikes [36]–[38]. Although the generality of this phenomenon among the RGCs is not well understood, we also investigated the effects of enhanced dendritic excitability on extracellular stimulation. While the threshold for stimulating the distal dendrites decreased with increasing dendritic I_Na_, the other regions (proximal dendrites, soma, AIS and axon) were minimally affected (Figure 2B1). The AIS consistently had the lowest threshold, even at the highest dendritic I_Na_ conductance tested (80 mS/cm^2^; Figure 2B2).

In summary, the location of lowest threshold was dictated by the AIS. This observation was invariant across the range of resistance inhomogeneity and dendritic excitability we examined. Therefore we only consider homogeneous tissue and nominal dendritic I_Na_ of 40 mS/cm^2^ in the remaining analyses.

### Stimulus Location Influences Activity Propagation through RGC Structures

**Figure 3:**
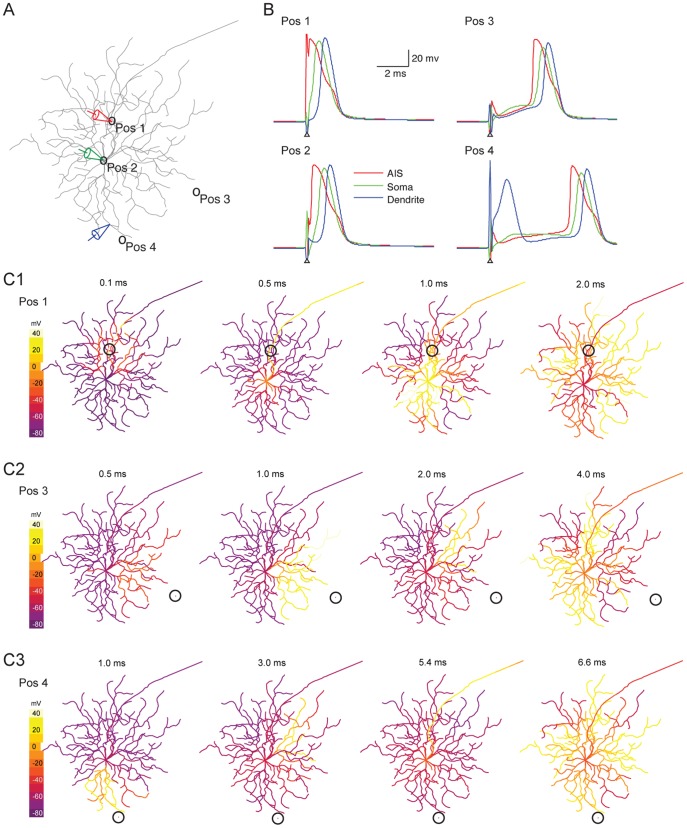
The effects of electrode placement on response propagation along cellular structures. (A) Recording electrodes were placed at the AIS (red), soma (green) and distal dendrite (blue) on the model Off RGC. Four stimulus locations were tested (pos1∼pos4). The stimulating electrode had 10 µm radius. (B) Intracellular responses at the AIS, soma and distal dendrite for the four stimulus locations. (C1– C3) Membrane voltage for the entire cell at discrete time points when stimulating at pos1 (C1), pos3 (C2) and pos4 (C3). The circles designate the electrode location.

The dendritic voltage-gated conductances influence neurons’ behavior under physiological conditions [39]. How does the depolarizing event propagate through the complex RGC structure following extracellular stimulation? We recorded the Vm of a model Off cell at the AIS, soma and distal dendrite (Figure 3A), while stimulating at the AIS, soma or one of two peripheral locations. The threshold stimulus was delivered through a 10 µm electrode. With the electrode over the AIS (position 1, Figure 3A), the stimulus elicited an AIS spike (latency  = 0.300 ms, Figure 3B), followed by a somatic spike (0.875 ms latency). Finally, a spike was observed in the distal dendrite 1.250 ms after stimulus presentation. A similar phenomenon was observed with the stimulating electrode over the soma (latency in ms, AIS = 0.550, soma = 1.200, distal dendrite = 1.575, Figure 3B). Stimulating at the cell’s periphery produced very different response profiles. At position 3, the stimulus elicited a small transient depolarization at all recording locations prior to full action potential (latency in ms, AIS = 2.950, soma = 3.450, distal dendrite = 3.750, Figure 3B). At position 4, the stimulus first elicited a dendritic spike, similar to those observed in RGC dendritic recordings [36]–[38]. Action potentials then occurred at the AIS, soma and dendrite, in that order (latencies in ms, AIS = 2.275, soma = 5.850, distal dendrite = 6.200). Notably, this configuration resulted in two dendritic spikes, a smaller initial spike and a larger back-propagating late spike. These four examples highlight the impact of stimulus location on RGC response profile following extracellular stimulation.

To investigate the dynamics of activity propagation at high spatial detail for the above stimulus positions, we examined the Vm of the entire cell at discrete time points. At position 1 (Figure 3C1), depolarization began at the AIS, then travelled orthodromically down the axon and antidromically towards the soma, before invasion throughout the dendrites. At position 3, (Figure 3C2) depolarization began at the dendrites closest to the stimulus, before propagating into the soma and axon. A spike was then generated at the AIS. Finally, the depolarization spread to dendrites distal to the stimulating electrode. At position 4 (Figure 3C3), depolarization also began at the dendrites closest to the stimulus. It then propagated toward the other neuronal elements. The resulting AIS spike antidromically evoked a somatic spike. Similar to back propagating action potentials (bAPs) observed in cortical neurons [40], the antidromic spike subsequently invaded the dendrites where the initial depolarization occurred, and elicited a second spike at this location. In summary, the active properties along the cellular structure strongly influenced RGCs’ response dynamics at the subcellular level following extracellular electrical stimulation.

### Threshold Map Changes with Electrode Size

**Figure 4:**
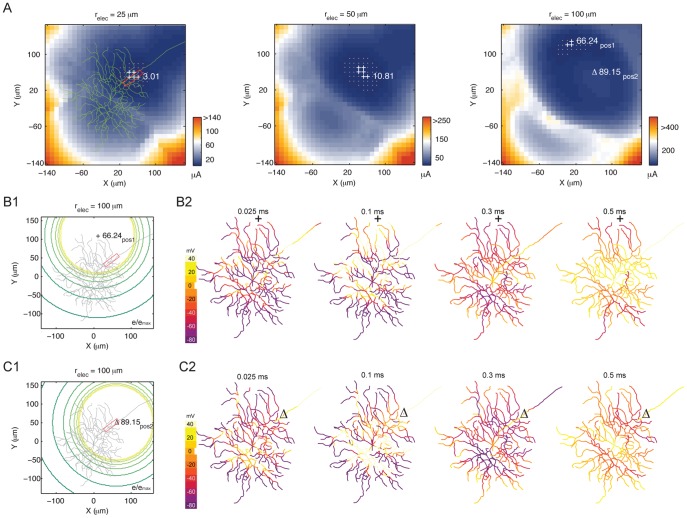
Threshold map and the migration of low threshold region with increasing electrode size. (A) Threshold map for 25, 50 and 100 µm radius electrodes. Red box, AIS. Number next to the ‘+’ and ‘Δ’ symbols is the threshold at that location. Dots, locations within 1% of the lowest threshold. (B1 and C1) Electric potential of the 100 µm electrode delivering threshold stimulus at pos1 (B1) and pos2 (C1). Each circular contour represents 10% relative change in field potential. Red box, AIS. (B2 and C2) Response initiation and propagation along the subcellular structures with the 100 µm stimulating electrode at pos1 (B2) and pos2 (C2).

A range of electrode sizes has been used clinically. We therefore examined the threshold map for other electrode dimensions (radius = 25∼100 µm, Figure 4A). The region with lowest threshold (marked by +) aligned with the AIS (red box). The dotted locations had threshold within 1% of the lowest value. With increasing electrode size, the region migrated away from the AIS.

To elucidate the reason for the changing threshold map with increasing electrode size, we inspected the electric potential generated by the electrode at position 1 and position 2. In Figures 4B1 and 4C1 the circular contour designates the normalized electric potential (e/e_max_). Each line represents 10% change. At both positions the electric potential is approximately constant around a region spanning roughly the electrode radius. However, for position 1 (Figure 4B1) the potential gradient changed rapidly in close proximity to the AIS, the most excitable element. Cellular depolarization began at these regions (Figure 4B2). In contrast, at position 2 (Figure 4C1) the electric potential was homogeneous along the proximal axon and the AIS. The regions with rapid potential change encompassed the dendrites beyond the soma and along the axon some 100 µm distal to the AIS. Response initiation began at these dendritic and axonal regions (Figure 4C2), which had higher threshold comparing to the AIS. Therefore, lowest threshold was produced by having the potential changing (the derivative) rapidly in close proximity to the AIS. This phenomenon was particularly prominent for large electrodes. Within the radial region of the electrode, the electric potential was approximately constant and only began to change rapidly at, and beyond, the electrode edge. In summary, the site of response initiation was strongly influenced by the location of potential gradient variation in relation to the neuronal elements, rather than the location of the electrode center *per se*.

### Low Threshold Hotspots in the RGC Mosaic Limits Multi-cell Activation

**Figure 5:**
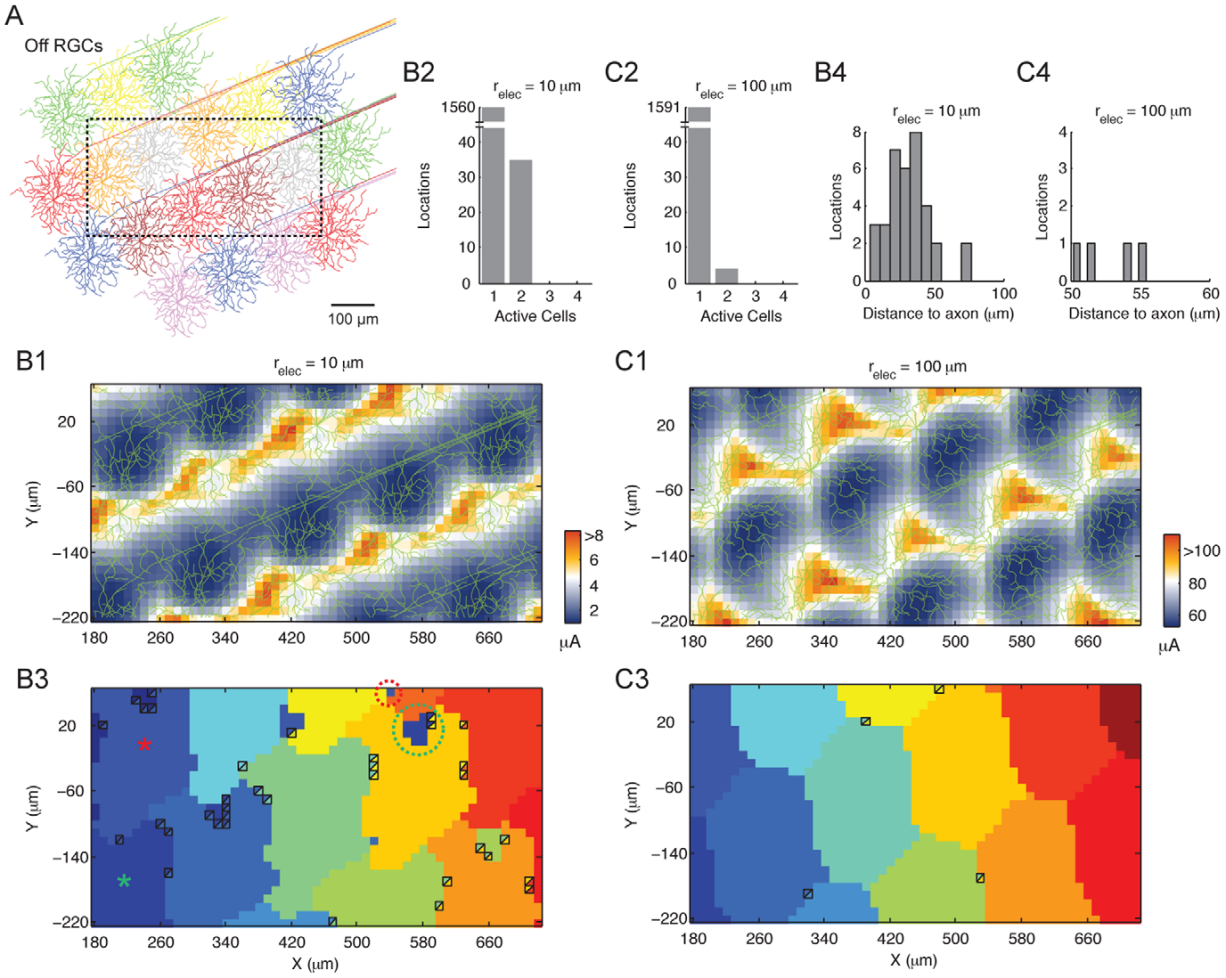
Responses of an Off RGC cluster following electrical stimulation. (A) A mosaic of Off cells. Boxed area denotes the stimulus test region. (B1 and C1) Threshold map for the test region for a small (10 µm; B1) and large (100 µm; C1) electrode. (B2 and C2) Histogram for the number of activated cells in the mosaic when stimulating at threshold in the test region for the small (B2) and large (C2) electrode. (B3 and C3) Identity map of activated cell(s) for each location when stimulating at threshold for the small (B3) and large (C3) electrode. The square boxes denote places with multi-cell activation. The colors in each box represent the identity of the active cells. At some locations (circled) the stimulus activated a cell several hundred µm from the electrode (*). Places with multi-cell activation (denoted by squares) tend to occur at positions of medium to high threshold, for both the small (B3) and large (C3) electrode. (B4 and C4) Euclidean distance of the multi-cell activation locations to the nearest axon, for the small (B4) and large (C4) electrode.

How do the RGCs respond to electrical stimulation as a population? We began by creating a mosaic of Off RGCs (Figure 5A) and examined the threshold map for a region (boxed in Figure 5A) within this mosaic for small (10 µm radius) and large (100 µm radius) electrodes. With the small electrodes, the lowest threshold region coincided with the locations of the AIS. The axons also formed a low threshold corridor (Figure 5B1). With increasing electrode size, the lowest threshold regions migrated away from the AIS (Figure 5C1). These observations are consistent with the single cell observations (Figure 4A). Finally, both maps contain repeated motifs of low threshold hotspots.

Although the threshold maps reveal neuronal excitability in space, they do not tell us how many nor which cells are activated when stimulating at threshold. These variables have direct impact on the psychophysical outcomes of retinal implants. We counted the number of active cells for each location in the test region (boxed in Figure 5A) following threshold stimulation. Here the threshold is defined as the minimum current that elicited a spike in one or more RGCs in the mosaic. In these analyses, all RGCs in the mosaic were considered, including those outside the test region. Furthermore, the RGC tiling extended beyond the electrode radii (hence the smaller test region in relation to the cell mosaic). Notably, threshold stimulation at most locations activated only one cell. This was true for both the small (Figure 5B2) and large (Figure 5C2) electrode. The stimulus activated at most two cells, but only rarely (2.2% with 10 µm electrode, 0.25% with 100 µm electrode). These results suggest limited neuronal activation when stimulating at threshold, irrespective of electrode size and despite the extensive passing axons.

We next identified the RGC(s) responsible for the responses at each location in the test region following threshold stimulation for the small (Figure 5B3) and large (Figure 5C3) electrode. Each cell is uniquely identified by color. The regions with a single active cell generally covered a large continuous area, which were spatially correlated to the low threshold hotspots (Figure 5B1 and 5C1). In Figure 5B3 and 5C3 the square boxes mark spots with two active cells. The colors within each box designate the active cells’ identity. These co-activation spots occupied places with moderate to high threshold; and were displaced from the axons with mean Euclidean distance of 31.7 µm and 52.7 µm for the small and large electrodes, respectively (Figure 5B4 and 5C4). Finally, co-activations were generally limited to neighboring cells, except for two locations (circled in Figure 5B3), where the stimulus activated a cell with soma several hundred µm away (* in Figure 5B3). In summary, the RGCs formed low threshold hotspots, thereby limited multi-cell activation and promoted localized recruitment when stimulating at threshold. In addition, the probability of multi-cell activation increased by stimulating at locations slightly displaced from the axonal trajectory, rather than directly over the axons.

### Similar Response Characteristics also Emerge with Different Mosaics and Cell Types

**Figure 6:**
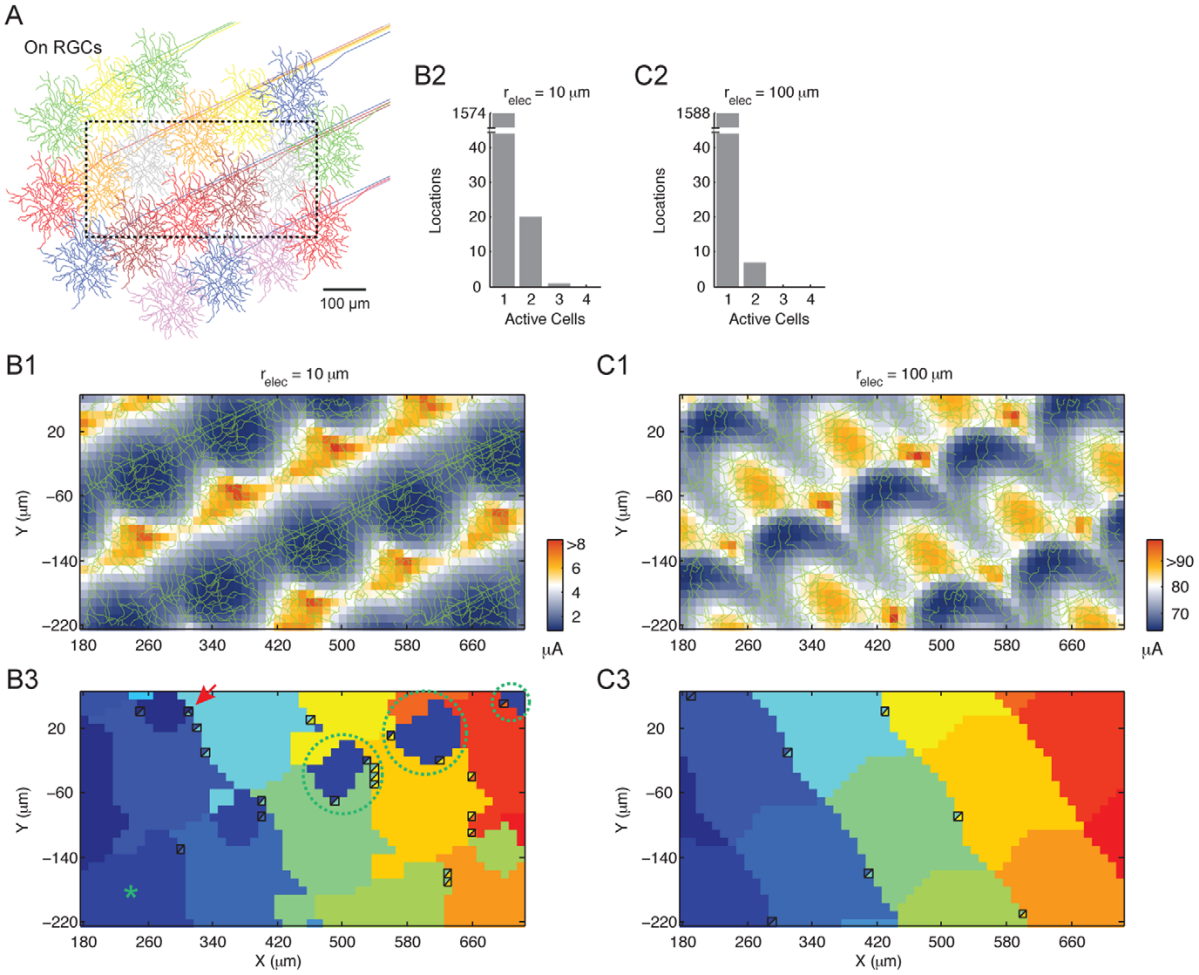
Responses of an On RGC cluster following electrical stimulation. See Figure 5 captions. Red arrow, threshold stimulus activated three cells at this position.

How robust are the above observations? We examined another mosaic (Figure 6A), with a slightly different coverage pattern and cell type (On RGCs). Consistent with the foregoing results, for both small (10 µm) and large (100 µm) electrodes the threshold map contained low threshold hotspots (Figures 6B1 and 6C1). Also paralleling the previous results, stimulating at threshold: (1) activated predominantly one cell (99.0% and 99.6% of all locations for small and large electrodes, respectively. Figure 6B2 and 6C2); (2) the regions with a single active cell generally covered a large continuous area (Figure 6B3 and 6C3); and (3) at restricted locations (circled in Figure 6B3) the stimulus activated cell with soma several hundred µm away (* in Figure 6B3). Thus limited multi-cell activation and localized recruitment were generally insensitive to cell types, cell arrangement and electrode size, when stimulating at threshold.

### Recruitment Profile of Supra-threshold Stimuli is Affected by Stimulus Location

**Figure 7:**
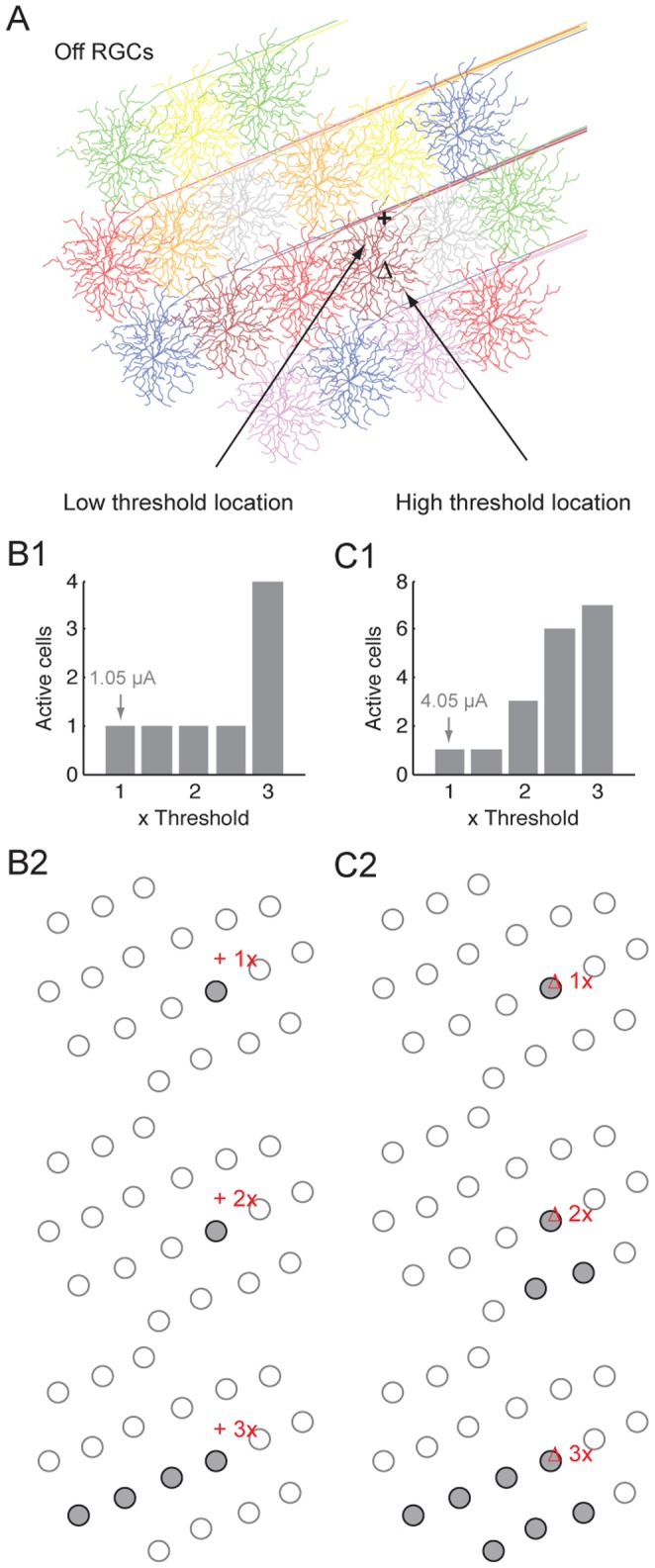
Responses of a RGC cluster to supra-threshold stimulation. (A) RGC mosaic and two stimulus locations, one had low threshold (+) and the other had high threshold (Δ). The electrode radius was 10 µm. (B1 and C1) The number of activated cells for the low threshold (B1) and high threshold location (C1) as a function of stimulus strength (1∼3 times threshold). Number (in µA) above each arrow denotes the threshold current at that location. (B2 and C2) Identity of the activated cell(s) in the cell mosaic (A), at 1x, 2x and 3x threshold current for the low threshold (B2) and high threshold (C2) location.

We used threshold stimuli in the foregoing analyses. How would a cluster of RGCs (of the same type) respond to supra-threshold stimuli? We conducted the analysis with a 10 µm radius electrode at two spots in the Off cell mosaic, one with low threshold (+ in Figure 7A) and another with high threshold (Δ in Figure 7A). The stimulus activated one cell at the low threshold spot, and continued to do so until 3x threshold current. At this current level, the number of activated cells abruptly increased to four (Figure 7B1). The additional cells recruited were activated via their passing axons in close proximity to the electrode. These neurons spanned a streak across the cell cluster (Figure 7B2). In contrast, at the high threshold spot the number of activated cells increased progressively with stimulus strength (Figure 7C1). The stimulus activated two neighboring cells before recruiting distant cells via their passing axons (Figure 7C2). These two examples illustrate that the number and spatial profile of neurons recruited by supra-threshold stimuli were profoundly affected by the stimulus location in the cell cluster. Some locations favored activation over extended space, while others favored localized activation.

### Midget Cell Response Profiles are Generally Comparable to Large-field RGCs

**Figure 8:**
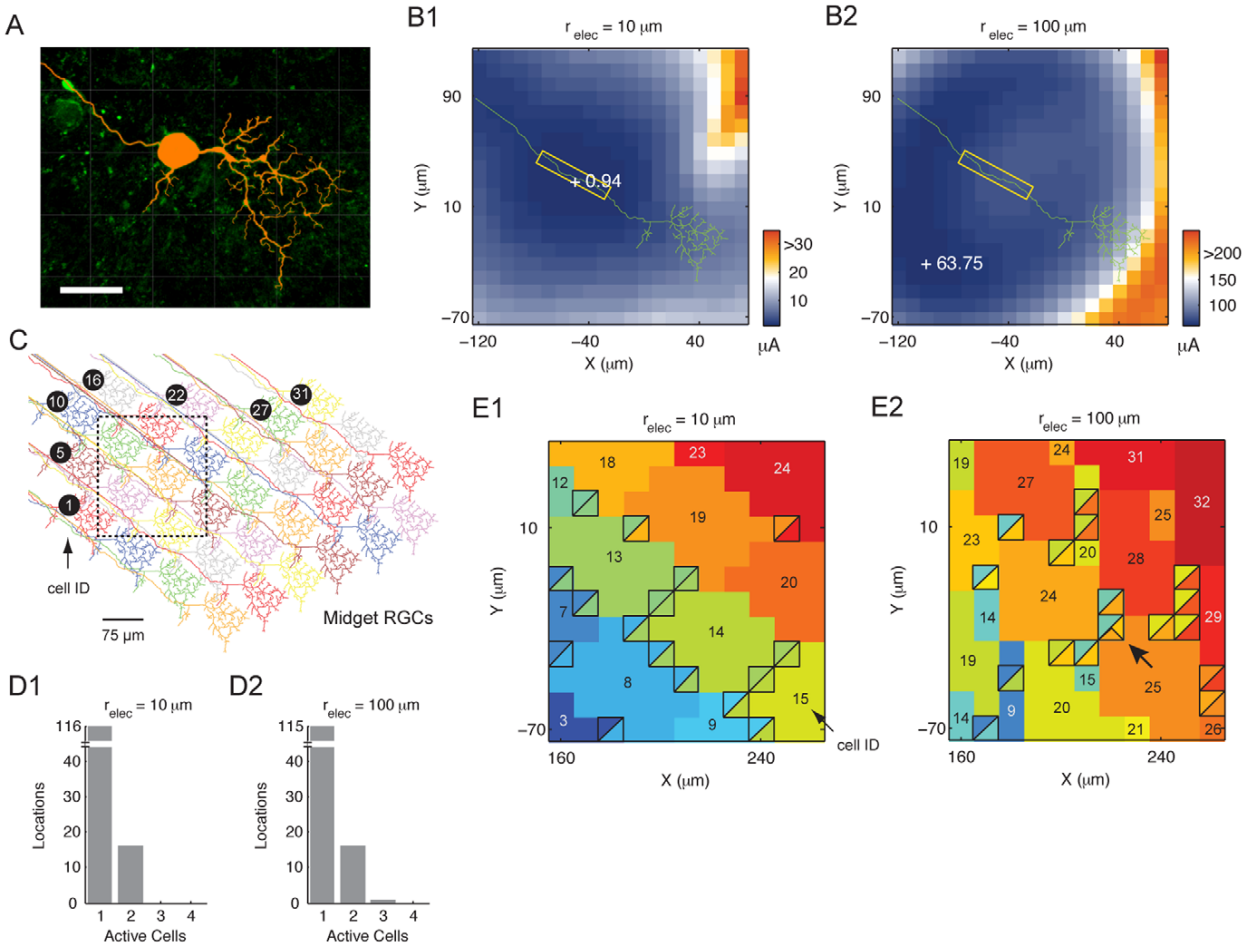
Responses of midget RGCs as a single-cell and as a cluster. (A) Traced midget cell volume overlaid on original confocal fluorescent image. Scale bar, 20 µm. (B1 and B2) Threshold map of the model midget RGC with small (10µm; B1) and large (100 µm; B2) stimulating electrodes. Number next to each+symbol is the threshold at the marked location. Boxed region, AIS. (C) A mosaic of 33 midget RGCs, with each cell uniquely identified by an ID. The boxed area indicates the test region. (D1 and D2) Histogram for the number of active cells with the small (D1) and large (D2) electrode. (E1 and E2) Identity map of activated cells following threshold stimulation in the test region for the small (E1) and large (E2) electrode. The square boxes denote places with multi-cell activation. The colors in the box designate the identity of the activated cells. The number in each color patch is the activated cell’s ID. Arrow, location with three activated cells.

Midget RGCs are unique to primates. These cells account for approximately 80% of the RGC population [41] and are critical elements of the image-forming pathway and for providing high acuity vision in human. These cells have small dendritic span (5∼100 µm; [42]) and are present with high spatial density near the fovea [43]. How might these characteristics affect their electrically evoked responses as a single-cell and as a population? We built a detailed midget cell computational model using the morphology of a marmoset monkey midget RGC (Figure 8A). We began by examining the cell’s threshold. For a small electrode (Figure 8B1; 10 µm), the region of lowest threshold aligned with the AIS (boxed region). With increasing electrode size (Figure 8B2; 100 µm), the lowest threshold region migrated away from the AIS. These observations are analogous to those of the large-field RGCs (Figure 4).

Next, we explored how a mosaic of midget RGCs responds to extracellular stimulation. We moved a disk electrode across a two-dimensional plane within a midget cell cluster (boxed region in Figure 8C). With threshold stimuli the small electrode (10 µm) activated one cell in the tested region, and very rarely, two cells (Figure 8D1). In the identity map, regions with one active cell covered large continuous areas (Figure 8E1). These features are comparable to those of the large-field RGC cluster. Due to the AIS displacement from the soma, the soma of most recruited cells was outside the test region. We also examined how the midget RGC cluster responded to a large electrode (100 µm). At most locations, threshold stimulation activated one cell, and occasionally two cells (Figure 8D2). Triple-cell activation only occurred at one location when stimulating with the large electrode (arrow in Figure 8E2). The large electrode also created a fragmented identity map and the somatic location of all activated cells was outside the test region (Figure 8E2).

## Discussion

In this study we found that at the single-RGC level: (1) the dendrites, despite their extensive coverage and total volume, always had higher threshold than the AIS and the axon; (2) the electric potential gradient in relation to the excitable neuronal elements, rather than the location of the electrode center, determined the threshold; (3) stimulus location strongly influenced the site of response initiation and the dynamics of subsequent activity propagation through the cell; and (4) these findings were robust with respect to tissue resistance inhomogeneity along the direction perpendicular to the electrode. For a population of RGCs we found that: (1) the RGCs formed low threshold hotspots, thus promoting focal activation when stimulating at threshold; (2) this phenomenon was robust with respect to electrode size, cell type and cellular arrangement; and (3) when stimulating with a supra-threshold stimulus, some locations favored activation over extended space, while other locations favored localized activation. We also developed the first framework for large-scale, parallel simulations of morphologically and biophysically detailed multi-cell models in the context of retinal extracellular stimulation. This may serve as a useful tool for future investigations.

### Comparison of Threshold Map to Previous Studies

The insensitivity of dendrites to extracellular stimulation here parallels previous findings in the brain and the spinal cord (e.g. [44]–[46]). Our threshold maps (e.g. Figure 1) are consistent with experimental findings of Fried [32] and simulation work of Jeng et al. [33]. An earlier threshold mapping study by Jensen [17] reported a more fragmented map, which is reminiscent of our results on midget RGCs stimulated with large electrodes. The RGCs were not identified in [17]. Based on the present findings, if some of the cells in Jensen’s study were small dendritic field RGCs, this may explain the inconsistency with Fried’s findings. Furthermore, the maps in [17] were averaged over many cells. The threshold profile was highly specific to the geometry of each cell in our study. Thus averaging may have further smeared out the threshold map.

### Direct RGC Stimulation Produces Variable Response Latencies

Experiments have reported a wide range of values for the latency of direct RGC stimulation (e.g. 0.25∼3.0 ms; [16], [18], [47], [48]). The results in this study suggest that this variability could be accounted for by the stimulus location in relation to the cellular structures. Stimulating the AIS produced the shortest latency, while stimulating the distal dendrites away from the axon trajectory tended to produce long latency responses.

### Electrode Position is a Poor Predictor of Response Origin

In our results the position of the stimulating electrode center was often a poor predictor for the location of neural response. Instead, the electric potential gradient in conjunction with neuronal element excitability was a better determinant of response origin and stimulus threshold. Large electrodes, due to the increased excitability beyond the electrode edge, further exacerbated this effect. This “edge-effect” (also called activation function [46], [49]) has also been suggested in the context of penetrating electrodes for deep brain stimulation [50]. Excluding the obvious cases of passing axon activation, these two factors together could cause signification discrepancy between the excited cell’s position and the electrode center location. This phenomenon may be an additional reason for the percept and implant electrode positional mismatches in human subjects [24].

### Large Electrodes do not Necessarily Recruit More Cells than Small Electrodes

It is generally thought that large stimulating electrodes will necessarily activate more cells, thus eliciting coarser percepts, and by using smaller electrodes finer percepts will be achieved. When stimulating at threshold, both small (10 µm radius) and large (100 µm radius) electrodes in our simulation activated very few RGCs of the same type. Indeed, threshold stimuli activated only one cell at most locations. Two observations could explain why large electrodes do not necessarily recruit more cells (of the same type). First, large variations of neuronal element excitability in space limit multi-cell activation. When stimulating at threshold, the current was just sufficient to activate the AIS or possibly the axon. Given the dramatic threshold increase with distance from these elements, the stimulus was generally insufficient to activate multiple nearby cells. Second, the relatively iso-potential region at the center of large electrodes was comparatively less effective at eliciting neuronal responses than the region at, and just beyond, the electrode edge (see Figure 4). Despite the electrode area increasing by the radius squared, the highly effective stimulus region beyond the electrode edge only increased linearly with radius. Therefore, as the electrode size increases, the highly effective stimulus region of the electrodes grows at a much slower pace than their coverage area.

### Limited Activation of Passing Axons with Threshold Stimulus

We observed limited passing axon activation in this study when stimulating at threshold. Generally, supra-threshold stimulation was necessary to cause extensive axonal activation. This may explain the disparity in experimental findings on the extent of axonal stimulation. Our results suggest that the absence of axonal activation, for instance reported by Sekirnjak et al. [18], [25], [51], was due to stimulating near threshold in close proximity to the AIS. Indeed, this was hypothesized in Sekirnjak et al. [25] through RGC receptive field and threshold mapping. In contrast, extensive passing axon activation seen in Behrend et al. [26] was likely a consequence of supra-threshold stimulation. Consistent with this interpretation, in order to elicit measurable calcium transients, stimulus trains evoking a dozen or more action potentials were necessary in that study [26].

### Predicting Responses in a RGC Mosaic with Multiple Cell Types and Superimposed Soma

We examined the RGC types individually in this study. How might a mixed-type RGC mosaic behave? RGC somas are also stacked over several layers in the central retina. How would this affect the RGCs’ responses? The present findings offer insights into these more complex conditions. In a multiple-RGC-type mosaic, the threshold for each neuron will still be determined primarily by the AIS. Thus activation will begin with cell types having the lowest AIS threshold. As the stimulus amplitude is increased, cell types with higher AIS threshold are increasingly activated. At the same time, progressively larger regions of the low-threshold RGCs are also recruited, in ways similar to the observations in Figure 7. The RGC neurites’ threshold to extracellular stimuli has the order: AIS<axon<soma<dendrites. This, together with the observation that the AIS and axon are always the elements closest to the epi-retinal electrode, means we could expect the AIS and the axon to be the primary determinant of RGC excitability even when the RGC somas are stacked over multiple layers.

### Implementation Considerations

An effective strategy for managing the complexity of large neural network simulation, and hence computation time, is to use a simplified representation for the dendrites [52]. Such reduced models are still able to capture important biological behaviors [29]. However, for electrical stimulation, the granularity at which the model neurons experience the extracellular electric potential is determined by the resolution of neurite representation. Detailed representation of neurons in space, as we have done here, is thus important for accurate predictions. Finally, we represented neuronal processes as 1-dimensional cables and the extracellular voltage profile across the cross-sectional area of the neuronal elements is assumed to be approximately constant. If the electrode size or the distance of the electrode to the cells is further reduced, this assumption may no longer hold, and the model will begin to lose accuracy.

We leveraged the power of high performance computing clusters to simulate morphologically and biophysically detailed RGC models, both as a single cell and as a mosaic. Mapping the threshold for a cluster of RGCs (e.g. Figure 5) took approximately 7.7 hours on a 128-core cluster. Running the same algorithm serially would take a prohibitive 17.5 days (423 hours). Implemented with a highly scalable architecture in NEURON and the MPICH2 interface, the technique could be used to study bigger and more complex neuronal networks by increasing the number of processor cores.

We have focused on the RGCs in this study. Following low-amplitude epiretinal stimulation, cellular activation is primarily confined to the RGCs [16], [18]. Thus the absence of presynaptic cells in the model would not drastically affect the present findings. Nevertheless, a logical extension is to consider cell types presynaptic to the RGCs. This would be particularly instructive for regimes such as subretinal [6] or suprachoroidal [53] stimulation, where the retinal network would likely contribute significantly to the total responses following stimulation. Finally, the intrinsic properties of RGCs may differ between types [54], [55] and these may change with retinal degeneration ([22]; but see also [56] for contrasting findings). If so, it would be instructive to incorporate these changes and investigate how they affect the RGCs’ responses to extracellular stimulation.

### Implications for Retinal Prosthesis Development

We found the stimulus amplitude to be the critical determinant for achieving focal activation. To conserve localized responses, it may be important for implants to use current at, or only slightly above, the threshold. Strong stimuli, even when delivered via small electrodes, caused extensive neuronal activation. Finally, when stimulating at threshold it may not be crucial to use small electrodes for the purpose of achieving focal stimulation at single-cell resolution (cell of the same type). Large electrodes also have the additional benefits of a higher charge injection limit and being more mechanically robust. However, the size of individual large electrode does limit the electrode array packing density and hence affects implant resolution.

## Materials and Methods

### Neuron Models

#### Geometry

We used Hodgkin-Huxley conductance-based multi-compartment models [31], [33] in all simulations. We examined two broad categories of RGCs, based on the size of their dendritic span. For large-field RGCs we used the morphology of mammalian On and Off RGCs traced from mice retinas [57]. The On cell was a Cluster 3 RGC with 196 µm average dendritic diameter and stratified at a depth of ∼40% in the inner plexiform layer (border of ganglion cell layer = 0%). The Off cell was a Cluster 2 RGC with 191 µm average dendritic diameter and stratified at a depth of ∼70% in the inner plexiform layer. These cells were chosen for their similarity to the primate parasol cells at ∼4 mm eccentricity from the center of the fovea in the temporal quadrant or ∼5 mm in the nasal quadrant [58].

The second category of RGCs examined was the small field midget cells unique to primates. We used midget cells from marmoset monkey (*Callithrix jacchus*) retinas. The technique for morphological reconstruction of these cells is described in the *Experimental procedures* section. The morphological data were digitized in *swc* format and subsequently imported into NEURON. For computational simulations, we chose a midget cell with dendritic span expected for cells at ∼4 mm eccentricity in the temporal quadrant [42].

Ensuring the axon extended well beyond the test area of the extracellular stimulating electrode, we linearly extended the axon of all RGCs by 900 µm, starting from the end point of the experimentally traced axon. Noting that the surface area of a cylinder with equal length and diameter is identical to the surface area of a sphere with the same diameter, we modeled the soma of each cell as a cylinder. For clarity, we omitted the width information in the Figures, and only illustrated the skeleton of the morphology.

#### Biophysics

The biophysics of all RGCs was modeled similarly. While the ionic current properties may differ between RGC classes [54], [55], limited information on current characterization and channel distribution in mice and primates precluded us from incorporating these details. Each neuronal compartment was endowed with a set of conductances to reflect the complement of ion channels that confer excitability properties to RGCs, as described in detail previously [31], [33]. The dendritic compartments contained transient voltage-gated sodium, delayed-rectifying potassium, A-type potassium, L-type calcium and calcium-gated potassium channels (in mS/cm^2^; gNa = 40, gK = 12, gA = 36, gCa = 2, gKCa = 0.05). The soma and axon hillock (first 50 µm of the axon) contained transient sodium, delayed-rectifying potassium, A-type potassium, L-type calcium and calcium-gated potassium channels (in mS/cm^2^; gNa = 70, gK = 18, gA = 54, gCa = 1.5, gKCa = 0.065). The axon initial segment (AIS) with high density of sodium channels [32] was located ∼50 µm distal to the somatic center and spanned 50 µm length. The AIS had identical ionic currents to the soma, except for higher sodium conductance (gNa = 700 mS/cm^2^). All other axonal sections had transient sodium, delayed-rectifying potassium and calcium gated potassium channels (in mS/cm^2^, gNa = 70, gK = 18, gKCa = 0.065). A non-specific voltage-gated leak current was present throughout the entire cell (gL = 0.005 mS/cm^2^). The reversal potential for sodium, potassium and leak was 35, −75 and −62.5 mV, respectively. The membrane capacitance and intracellular axial resistance for the cells were 1 µF/cm^2^ and 110 Ω cm. To examine RGC dendritic excitability following extracellular stimulation, we also varied dendritic I_Na_ over a range of values (40∼80 mS/cm^2^).

The number of model segments affects the spatial granularity at which the RGCs experienced the extracellular electric potential. We ensured the length of every segment was <12 µm. We ascertained the adequacy of this granularity by tripling the segment number then checking the model still produced comparable results.

### Cell Calibration

**Figure 9:**
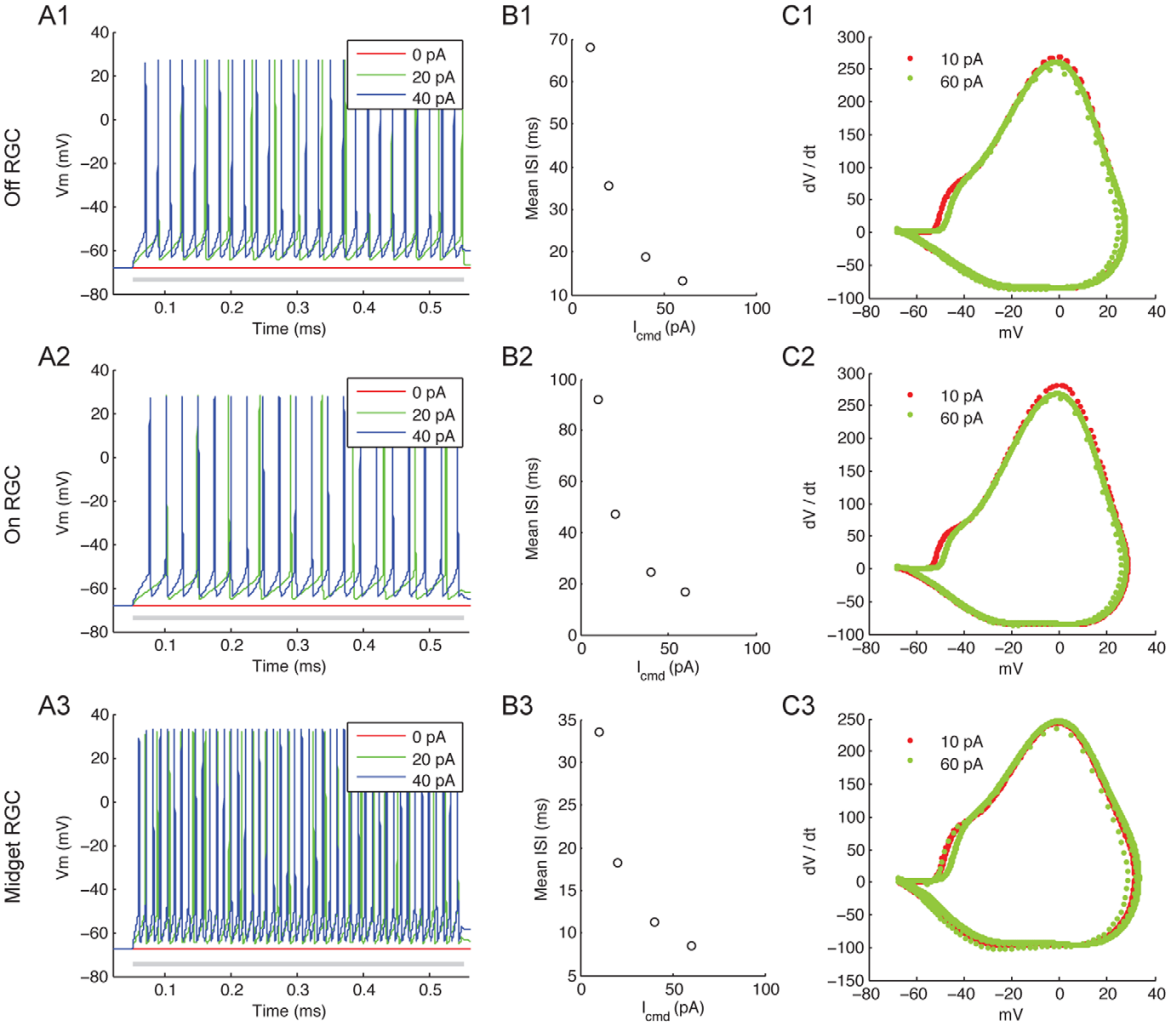
RGC responses evoked by intracellular current injection. All intracellular current pulses were applied for 500 ms. (A1∼A3) Vm responses of the Off, On and Midget RGC to 0, 20 and 40 pA depolarizing current. Gray bar, duration of current injection. (B1∼B3) Mean inter-spike intervals during depolarizing current injection. (C1∼C3) Response phase portrait for 10 and 60 pA current injection.

To ensure behavioral consistency of the RGC models to previous work [31], we examined the cells’ spiking responses to depolarization by intracellular current injection (Figure 9A), mean inter-spike intervals during current injection (Figure 9B), and the phase portraits of the spiking responses (Figure 9C). Notably, despite identical biophysics specifications, morphological variations were sufficient to produce different behaviors among the cells. This is in agreement with [30].

### Cell Cluster Models

To create a RGC cluster, we tiled multi-compartment RGCs, as described above, over a two-dimensional plane, initially with deterministic distance between neighboring cells. We then introduced spatial randomness by jittering each cell’s location by Gaussian distribution with 5 µm standard deviation. For the small field midget cells we used 1 µm standard deviation. Spatial coverage for the RGC can be quantified precisely by a dimensionless coverage factor [59]:
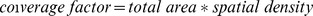



Incomplete coverage is indicated by values less than 1, a coverage factor of 1–3 indicates some overlap, while greater values suggest extensive overlap. The coverage factors for the large-field 19-cell Off and On RGC mosaic in Figures 5 and 6 were 1.28 and 1.22, respectively. The midget RGC mosaic in Figure 8 had a coverage factor of 0.90. These values are in agreement with the characteristics of these cell types [60], [61].

### Electrical Stimulation

Unless noted otherwise, we considered the tissue medium to be an isotropic ohmic conductor [62]. The stimulation electrode was monopolar and purely resistive [34], [45], [63], [64]. Thus the voltage at a particular point in space can be calculated in a fashion similar to [44]:
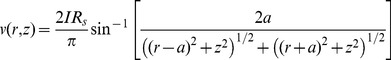
(1)where *I*, *a*, *r* and *z* are the applied current, electrode radius, radial and axial distance from the center of the disk electrode, respectively. *R_s_* is the electrode transfer resistance. We calibrated *R_s_* from experimental results [18]. Specifically, with experimentally derived stimulus threshold (*I*), electrode radius (*a*) and placements (*r* and *z*), *R_s_* is the minimum value required for eliciting an action potential. We used *R_s_* = 0.725 Ω for a 10 µm radius platinum electrode 40 µm above the cell. The transfer resistance for other electrode sizes could be calculated through surface area scaling relative to the 10 µm electrode. To stimulate the RGC(s), a charge-balanced, cathodic-first, biphasic pulse was delivered from the disk electrode at the vitreous-side (epiretinal stimulation) 40 µm axial distance above the RGC somatic center. Each phase of the biphasic pulse was 0.1 ms. No inter-phase delay was used.

The response latency is defined as the duration between stimulus onset and the action potential peak. The threshold is defined as the minimum current that evoked a somatic action potential. To map the threshold current as a function of position, we moved the disk electrode in the X-Y plane in 10 µm increments, and 40 µm above the somatic center. The threshold current was determined by binary search to within 0.1 µA resolution for the 10 µm radius electrode and within 0.5 µA for all other electrode sizes. Spikes were detected by 0 mV threshold crossing. All electrode movement, spike detection and threshold searching were performed online automatically by custom-written procedures.

### Inhomogeneous Tissue Resistivity

The above formalism for extracellular stimulation assumes homogeneous tissue [62]. The resistivity of the cell body layer could be approximately twice that of the dendritic and axonal layers when measured at high spatial resolution [34], [35]. We also examined how such inhomogeneity might influence the neural responses. Given the planar retinal architecture, we achieved this by multiplying *R_s_* in equation (1) with an axial-distance-dependent scaling coefficient *C(z)*:
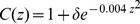
(2)


The profile of *C*(*z*) captures the characteristics observed in López-Aguado et al. [35]. The resistivity peaks at *z* = 0 µm (center of the somatic layer), and falls with distance from this region. To simplify presentation in Figure 2, we denote the resistivity at the center of the somatic layer and some arbitrarily far away z-direction position as R_peak_ and R_distant_, respectively. Furthermore, R_peak_ corresponds to R_s_ in Equation 1. The value *δ* determines the difference between maximum resistivity (at the center of RGC somatic layer) and minimum resistivity (distant to the somatic layer). Greater inhomogeneity is achieved by increasing *δ*.

### Computation on High Performance Computing Clusters

We implemented the models in NEURON versions 7.1 and 7.2 [27], [65], which we re-compiled from source for parallel computing support on Mac OS X and Linux. Simulations were carried out on Amazon Elastic Compute Cloud HPC (high performance computing) clusters. Each node had two eight-core Intel Xeon processors and 60.5 Gb of memory. Inter-node communication took place via the MPICH2 message-passing interface over a 10 Gigabit network. We typically used a cluster consisting of 128∼258 cores. We developed Python and Bash scripts for online management of the cluster.

To set up the cluster computing environment, we: (1) began with a EC2-specific Linux distribution, (2) created a temporary disk space; (3) installed GNU programming toolchain, MPICH2 and other dependency libraries; (4) compiled NEURON from source for parallel computing support and without user interface; (5) installed the resulting binary files; and (6) configured the user accounts. We then created an image of the temporary disk space and stored it permanently on Elastic Block Store (EBS). A snapshot of the machine was also created and stored as a permanent disk image.

### Parallel Computation

When computing the threshold map, we divided up the search space and ran the threshold searching procedure for multiple locations in parallel. This was achieved by NEURON’s *ParallelContext* object in conjunction with the MPICH2 Message Passing Interface library. Every simulation on each core ran independently. Every simulation process reported back to the master process prior to termination, with information on whether spikes were evoked, and if so, the spike latency and the stimulus amplitude. It is possible to load balance each simulation across multiple cores. However, because a single core could complete each simulation reasonably quickly, the extra latency and communication overhead involved in multi-core load balancing was not warranted.

The simulation time step was 0.025 ms at 35°C. Computation time for a threshold map of the 19-cell RGC cluster shown in Figure 5 (approximately 10,100 segments) took approximately 7.7 hours on a cluster containing 128 processor cores. We analyzed all results in Matlab R2010b (Mathworks).

### Computing Threshold Values

To determine the stimulus threshold for a particular electrode position, we ran the model (single cell or RGC cluster) for 40 ms. The stimulus amplitude was either increased or decreased by a binary search procedure. This algorithm has an average runtime complexity of O(log n). To create a threshold map, we repeated the threshold searching procedure over a two-dimensional plane at 10 µm resolution.

### Experimental Procedures

The marmoset (*Callithrix jacchus*) eyes used in this study were kindly donated by Dr Sam Solomon (Discipline of Physiology, The University of Sydney). The eyes were enucleated and hemisected behind the ora serrata, the lens was removed and the vitreous body was drained. The remaining eyecup containing the sclera, pigment epithelium and retina were transferred to a dish containing carboxygenated (95% CO_2_+5% O_2_) Ames medium. The remaining dissection procedures were carried out under infrared light. A small piece of the retina was isolated and transferred photoreceptor-side down into an imaging chamber perfused with Ames’ Medium at ∼5 mL/min and heated to 34∼35°C. We filled the cells with a whole-cell patch clamp electrode containing (mM): 120 KMeSO_4_, 10 KCl, 0.008 CaCl_2_, 0.5 EGTA, 1 MgCl_2_, 10 HEPES, 4 ATP-Na_2_, 0.5 GTP-Na_3_, 8 Neurobiotin-Cl and 0.075 Alexa Fluor 488, pH 7.2 (resistance 3.5∼5 MΩ). Neurobiotin loading occurred by passive diffusion for ≥20 min. The whole-mount retinas were fixed with 4% paraformaldehyde in phosphate buffer, incubated in PBS with 0.5% Triton-X and 1% BSA, reacted against Streptavidin - Alexa Fluor 488 conjugate, counterstained with DAPI, then mounted with Pro-long Gold. Sigma Aldrich, Invitrogen and Vector Laboratory supplied all chemicals and reagents. All procedures were approved and monitored by the Animal Welfare and Ethics Committee at the University of New South Wales and University of Sydney. We imaged the whole-mounts on a confocal microscope with a 20×0.7 NA air and a 40×1.1 NA oil immersion objective lens, then analyzed and traced the morphology with Imaris (Bitplane AG) and Fiji (National Institute of Health, USA).
